# Enhancing Multi-Label Chest X-Ray Classification Using an Improved Ranking Loss

**DOI:** 10.3390/bioengineering12060593

**Published:** 2025-05-31

**Authors:** Muhammad Shehzad Hanif, Muhammad Bilal, Abdullah H. Alsaggaf, Ubaid M. Al-Saggaf

**Affiliations:** 1 Department of Electrical and Computer Engineering, King Abdulaziz University, Jeddah 21589, Saudi Arabia; meftekar@kau.edu.sa (M.B.); usaggaf@kau.edu.sa (U.M.A.-S.); 2 Unit of Allergy and Immunology, Department of Pediatrics, King Abdulaziz University, Jeddah 21589, Saudi Arabia; ahmalsaggaf@kau.edu.sa

**Keywords:** multi-label classification, chest X-ray, convolutional neural networks, learning by ranking

## Abstract

This article addresses the non-trivial problem of classifying thoracic diseases in chest X-ray (CXR) images. A single CXR image may exhibit multiple diseases, making this a multi-label classification problem. Additionally, the inherent class imbalance makes the task even more challenging as some diseases occur more frequently than others. Our methodology is based on transfer learning aiming to fine-tune a pretrained DenseNet121 model using CXR images from the NIH Chest X-ray14 dataset. Training from scratch would require a large-scale dataset containing millions of images, which is not available in the public domain for this multi-label classification task. To address class imbalance problem, we propose a rank-based loss derived from the Zero-bounded Log-sum-exp and Pairwise Rank-based (ZLPR) loss, which we refer to as focal ZLPR (FZLPR). In designing FZLPR, we draw inspiration from the focal loss where the objective is to emphasize hard-to-classify examples (instances of rare diseases) during training compared to well-classified ones. We achieve this by incorporating a “temperature” parameter to scale the label scores predicted by the model during training in the original ZLPR loss function. Experimental results on the NIH Chest X-ray14 dataset demonstrate that FZLPR loss outperforms other loss functions including binary cross entropy (BCE) and focal loss. Moreover, by using test-time augmentations, our model trained using FZLPR loss achieves an average AUC of 80.96% which is competitive with existing approaches.

## 1. Introduction

Chest radiography is extensively utilized for identifying and diagnosing thoracic diseases. In healthcare facilities, radiologists rely on their expertise to visually examine these radiographs and report their findings. Recently, automated medical image analysis has emerged as a promising field, offering significant support to healthcare institutions in managing high patient volumes [1,2,3]. This progress has been made possible by remarkable advancements in areas such as computer vision, pattern recognition, and machine learning.

We have addressed the challenging task of the classification of thoracic diseases on chest X-rays (CXR) in this article. It is regarded as a multi-label classification (MLC) problem as multiple diseases can be found in a patient’s CXR. The task is challenging primarily due to the widely recognized long-tail distribution of disease occurrences as all diseases are not occurring with the same frequency in the dataset. Moreover, some diseases are also rare resulting in a class imbalance. In recent years, the problem has been a focus among computer vision and biomedical communities to build efficient deep learning-based models and associated training schemes to handle class imbalance [4,5,6,7,8]. Although the localization of diseases in CXR images is another related task alongside disease classification, this article focuses solely on the latter with emphasis on handling class imbalance.

Deep learning-based models have demonstrated outstanding performance in image recognition tasks within large-scale benchmark datasets such as ImageNet-1K [9] and ImageNet-21K [10]. The transfer learning technique is commonly employed to adapt models trained on these datasets to new tasks, including object detection [11], face recognition [12], image retrieval [13], image matching [14], medical image analysis [15,16,17], etc. These research works have shown that models using transfer learning outperform traditional machine learning methods in tasks they were not specifically trained for. It is important to note that the large-scale image recognition benchmark datasets like ImageNet-1K and ImageNet-21K associate a single label with each image, resulting in single-label prediction problems. Furthermore, these datasets typically feature balanced label distribution. Thus, the overall training scheme must be carefully designed to handle image classification tasks in the medical sector such as classifying thoracic diseases using CXR, which is a multi-label problem and also demonstrates class imbalance.

Over the past decade, significant efforts have been made to create benchmark datasets for detecting thoracic diseases in CXR images. In this regard, the NIH Chest X-ray8 dataset [4] was introduced in 2017, initially featuring labels for eight distinct thoracic diseases. This dataset was later expanded by the same authors to include 14 disease labels. It comprises 112,120 CXR images and is known as the NIH Chest X-ray14 benchmark dataset. Similarly, the CheXpert dataset [18], introduced in 2019, also includes 14 disease labels but contains a total of 224,316 CXR images. In the same year, the MIMIC-CXR dataset [7] was proposed, encompassing 377,110 images and 26 disease labels. Among these datasets, the NIH Chest X-ray14 is widely recognized for its usage, yet it remains particularly challenging due to its relatively smaller number of CXR images compared to the other two datasets. It is evident that these benchmark datasets are significantly smaller in scale than ImageNet-1K (1.2 million images) and ImageNet-21K (14 million images), which are commonly utilized to train deep networks. Consequently, transfer learning is the predominant approach for classifying thoracic diseases in CXR images [4,5,6,18,19,20]. For this very reason, it is common in the literature to find various well-established convolutional neural network architectures, such as ResNet [21], DenseNet [22], EfficientNet [23], MobileNet [24], Inception [25], and Xception [26], etc., being fine-tuned for this task. To address MLC, the sigmoid is commonly utilized in the final layer as the activation function, and the neural network is trained using binary cross-entropy (BCE) loss or its variants.

In this article, we also adopt transfer learning to fine-tune a pretrained densely connected convolutional network, specifically, DenseNet121. Contrary to the BCE loss, we have employed a rank-based loss to fine-tune the network. Specifically, we have modified the Zero-bounded Log-sum-exp and Pairwise Rank-based (ZLPR) loss function, proposed by Su et al. [27] for MLC tasks, to focus on hard examples during training, which are mainly instances of rare classes or labels. This is achieved by incorporating a temperature parameter in the loss function. As a result, the loss function is suited to addressing class imbalance effectively. We have drawn inspiration from the focal loss [11] in our methodology to modify the ZLPR loss. For this very reason, we call it the focal ZLPR (FZLPR) loss function. Experimental results on the NIH Chest X-ray14 dataset demonstrate that the proposed loss function surpasses both BCE loss and focal loss, and addresses class imbalance effectively. Similar to [6], we also employ test time augmentations as they are known to improve the classification accuracy. The following summarizes the main contributions of our work.

- The focal ZLPR (FZLPR) loss function is proposed by modifying the ZLPR loss function and incorporates a temperature parameter to effectively handle hard examples during training.
- Our experimental results demonstrate that the FZLPR loss function is effective in classifying thoracic diseases in CXR images. It outperforms the BCE loss and focal loss in terms of average AUC.
- By utilizing test-time augmentations, our model trained with the FZLPR loss achieves an average AUC of 80.96%, which is comparable to that of state-of-the-art methods.

The rest of article is organized as follows: Section 2 provides a review of existing techniques and methods proposed for disease classification in CXR images and loss functions for MLC tasks. Section 3 details the proposed methodology. The experimental results along with the implementation details are presented in Section 4. Finally, conclusions and future works are discussed in Section 5.

## 2. Literature Review

MLC is a focus of interest of researchers in the domains of natural language processing, computer vision, and medical image analysis. Numerous deep learning-based techniques, focusing on network architectures, loss functions, and training schemes, have been proposed in the literature. Below, we present a concise review of the literature on disease classification in CXR images, along with an overview of various loss functions proposed to handle class imbalance.

### 2.1. Multi-Label Classification for CXR Images

In a foundational work [4], pretrained models such as AlexNet, VGG-16, GoogLeNet, and ResNet50 are employed as feature extractors. The extracted features are fed into a transition layer (a convolutional layer) and a dense final classification layer, both of which are trained using weighted BCE loss for disease classification in chest X-ray (CXR) images. For disease localization, weighted spatial activation maps—acting as disease likelihood maps for each class—are generated by combining the outputs of the transition layer and the final classification layer. This approach achieved promising results on the NIH Chest X-ray8 dataset. In a related work [18], Irvin et al. investigated various convolutional neural network (CNN) architectures, including Inception-V4, ResNeXt101, ResNet152, and DenseNet121, using their proposed CheXpert dataset containing 14 distinct disease labels. The models were trained using a masked BCE loss function. Among the architectures evaluated, DenseNet121 demonstrated superior performance. Similarly, transfer learning is employed in [28] to fine-tune a pretrained EfficientNet on the NIH Chest X-ray14 dataset using BCE loss. The researchers utilized a non-official split of the dataset and achieved state-of-the-art results. In [29], a fine-grained framework trained using a combination of loss functions including multi-label Softmax loss, BCE loss, and label correlation loss, is proposed. ResNet101 and DenseNet121 are employed as backbones in the experiments using the NIH Chest X-ray14 dataset. They demonstrate that ResNet101 and DenseNet121 models trained with their proposed combined loss function achieve better performance compared to those trained with BCE loss alone. Baltruschat et al. [30] employed non-image data in addition to CXR images for disease classification. In addition to ImageNet-1K pretrained ResNet models, they developed custom ResNet models by varying the model depth. These models were trained from scratch using BCE loss on the NIH Chest X-ray14 dataset. Their findings indicate that while ImageNet-1K pretrained models perform adequately, a custom 38-layer ResNet model trained on CXR images and non-image data outperforms the others. Katona et al. [31] studied the impact of hyperparameters on fine-tuning the pretrained VGG16, ResNet50, and DenseNet121. A top network in the form of dense layers is added for the classification purpose. Experiments were conducted by varying batch sizes between 16 and 64, as well as exploring different optimizers during training using the NIH Chest X-ray14 dataset. Both BCE loss and focal loss were studied in their experiments. The results demonstrated consistent performance across all network architectures regardless of batch size, with the stochastic gradient descent (SGD) optimizer outperforming Adam and RMSprop. Moreover, BCE resulted in superior performance compared to the focal loss.

A category-wise residual attention learning (CRAL) framework is proposed in [20] for prediction of diseases in CXR images. In the first stage, a pretrained CNN backbone (ResNet50 or DenseNet121) is employed to extract features. The feature maps thus extracted are passed to an attention module composed of convolutional layers and residual connections. The purpose of attention module is to learn attention scores to weight different spatial positions of the feature maps. The CRAL is trained using BCE loss function in an end-to-end manner. Promising results are obtained on the NIH Chest X-ray14 benchmark dataset. In a complementary work, Han et al. [32] propose a knowledge-augmented contrastive learning framework for classification and localization of diseases in CXR images. Knowledge in the form of radiomic features is extracted with the help of Gradient-weighted class activation mapping (Grad-CAM) images and is combined with the image features extracted from a CNN-based image encoder—a pretrained ResNet18 model—in the proposed contrastive learning framework. Experimental results on the NIH Chest X-ray8 benchmark dataset show the efficacy of the proposed framework. In a similar work [19], an EfficientNet-B0-based detection and classification model is proposed for classification and localization of chest diseases in CXR images. In the proposed model, EfficientNet-B0 is utilized for feature extraction, followed by a feature network that performs feature blending in both top-down and bottom-up manners. A final detector then outputs the localization results with class labels. The results are presented on the NIH Chest X-ray8 benchmark dataset containing eight chest diseases only. An ensemble approach is proposed in [5] where features extracted from multiple CNNs are concatenated and passed through a dense layer for final prediction. The ensemble is trained with the BCE loss function using the MIMIC-CXR benchmark dataset. The experimental results on the NIH Chest X-ray14 benchmark dataset are presented using two distinct ensembles employing different CNN architectures like InceptionV3, MobileNet, DenseNet121, Xception, RegNet-X, and ResNet50. It is shown that the ensemble composed of InceptionV3, ResNet50, and DenseNet121 outperforms other state-of-the-art methods.

In general, ImageNet-1K pretrained models handle images of size 224 × 224 pixels. In the aforementioned works, this resolution is used to fine-tune models. However, high-resolution CXR images are also employed for disease classification in the literature. Guendel et al. [33] used 1024 × 1024 pixel images (more than four times the size of ImageNet-1K images) as inputs to train a DenseNet121 model. To accommodate the larger input size, two convolutional layers are added before DenseNet121, as the pretrained model can handle an input size of 224 × 224 pixels. This results in a highly compute-intensive model. Their experimental results showed that high-resolution CXR images were beneficial for disease classification on the NIH Chest X-ray14 dataset. In [6], EfficientNetV2 and ConvNeXt models, which can process large image sizes, were employed. Their model uses 768 × 768 pixel image as inputs. During training, several image augmentation techniques are employed to handle the class imbalance. Additionally, focal loss [11] is employed to handle the long-tail distribution of classes. The proposed method achieves a top-five ranking in the CXR-LT competition [8] on the MIMIC-CXR dataset.

### 2.2. Loss Functions for Class Imbalance

In non-trivial classification tasks like MLC, handling class imbalance effectively is highly desired but is challenging. The issue is not confined to the medical image classification tasks but extends to other research areas such as natural language processing and computer vision. The weighted BCE loss is frequently employed for MLC due to its simplicity. However, the class frequency based re-weighting scheme performs poorly [34,35]. Focal loss [11] was introduced in the context of objection detection tasks and aims to focus on hard examples through a dynamic re-weighting scheme during training. It has demonstrated superior performance compared to BCE loss. Asymmetric loss [36], inspired by focal loss, was proposed for MLC that uses different re-weighting scheme for positive and negative examples. The results on various computer vision tasks including object detection demonstrate that the asymmetric loss performs better than the focal loss. Bénédict et al. [37] propose SigmoidF1 loss that approximates the macro-F1 score for MLC. This differentiable loss function enables end-to-end model training using stochastic gradient descent. Experimental results on both text and image datasets demonstrate its superior performance compared to BCE, focal, and asymmetric loss functions. Huang et al. [38] applied Taylor series expansion to BCE loss and proposed a generalized asymmetric loss called asymmetric polynomial loss (APL). To address class imbalance, APL controls the gradients of positive and negative examples on a term-by-term basis within the loss function. While it has shown to outperform BCE and asymmetric loss on multi-label text and image classification tasks, it requires tuning a large number of hyperparameters. In a follow-up work [39], the authors proposed controlling the contribution of negative label gradients by adding a regularization term in APL loss. Experimental results on multi-label classification tasks demonstrate the effectiveness of this regularization compared to BCE, focal, and asymmetric loss functions. A similar approach is employed in [40], where balanced asymmetric loss (BAL) is proposed. BAL prioritizes hard positive examples during training and, when combined with the contrastive loss from contrastive language-image pre-Ttraining (CLIP), has been shown to outperform various loss functions, including focal and asymmetric losses, in long-tail multi-label classification tasks.

Ranking-based losses have also been discussed extensively in the literature for MLC. Among them, LSEP [41] is a pairwise ranking-based loss function that serves as a smooth approximation to the widely known hinge loss. The smoothness is achieved by utilizing the log-sum-exp function. It is shown that the LSEP delivers superior ranking performance compared to the standard pairwise ranking loss. However, unlike BCE and focal loss functions, LSEP lacks a built-in label decision mechanism. To address this, a separate method for determining the threshold for each class is introduced in [41]. In a follow-up work [27], Su et al. proposed a fixed threshold for LSEP and formulated a novel loss function called Zero-bounded Log-sum-exp and Pairwise Rank-based (ZLPR). The empirical results show that the proposed ZLPR loss handles label dependencies and ranking between positives and negatives effectively and performs better than LSEP, BCE and focal losses on text and image benchmark datasets. Recently, Ref. [42] proposed the hierarchy-aware biased bound margin (HBM) loss, conceived to modify ZLPR by incorporating learnable bounds, biases, and margins to address the limitations of fixed thresholds. Experimental results on text classification tasks reveal that HBM performs marginally better than ZLPR. Distributionally robust (DR) loss, proposed in [43], is based on class-wise LSEP loss. It incorporates a gradient constraint for negative labels to regulate their influence during training, as they constitute the majority and can disproportionately affect the LSEP loss, favoring negative labels. Experimental results on long-tail multi-label classification tasks show the effectiveness of DR loss compared to various loss functions, including LSEP and focal loss.

Based on the reviewed literature, it can be concluded that networks trained on ImageNet benchmark datasets are well-suited for thoracic disease classification in CXR images by employing transfer learning. Moreover, DenseNet121 has emerged as a promising architecture, frequently adopted in numerous research works, and has achieved superior performance compared to its counterparts. Furthermore, recent advancements in ranking-based loss functions demonstrate their potential in handling non-trivial classification tasks such as MLC. The methodology described in the following section is inspired by these insights from the literature.

## 3. Materials and Methods

Let $S={\left\{\left(x_{n},L_{n}\right)\right\}}_{n=1}^{N}$ be a multi-label dataset consisting of *N* image-label pairs where $x_{n}$ represents the $n^{th}$ image in the dataset and $L_{n}$ denotes its corresponding set of possible labels. Let L={1,2,3,…,M} be the set of all labels, totaling *M*. Then, $L_{n}\subseteq L$ and each image is associated to a different number of labels. The objective is to train a multi-label classifier $f(x_{n},\theta )$ with θ as learnable parameters that predicts all possible labels ${\hat{L}}_{n}$ for a given input image $x_{n}$. In this work, the f(·,θ) is modeled as a CNN model that learns labels predictions from input images in an end-to-end manner using an appropriate loss function. The specifics of the loss function and CNN architecture are detailed in the following sections. In general, the function f(·,θ) outputs a vector of unbounded real numbers called logits or label scores, which can be transformed into a probability using the sigmoid function. Let $f_{m}\left(\cdot ,\theta \right)$ be the predicted score corresponding to *m*th label ${\hat{l}}_{n}^{m}\in {\hat{L}}_{n}$; we can write

(1) $${\hat{l}}_{n}^{m}=\frac{1}{1+e^{-f_{m}\left(x_{n},\theta \right)}}.$$

### 3.1. Focal ZLPR Loss

For accurate classification, it is desired for $f(x_{n},\theta )$ to yield higher values for labels that are associated with the image $x_{n}$ compared to those that are not. Consequently, each image is accompanied by both positive labels, $L_{n}^{pos}$, and negative labels, $L_{n}^{neg}$. The LSEP loss function [41] employs pairwise comparisons to achieve higher label scores for positive labels compared to negative labels. Mathematically, we can write the LSEP function as

(2) $$\mathrm{LSEP}=log\left(1+\sum_{j\in L_{n}^{neg}}e^{f_{j}\left(x_{n},\theta \right)}\sum_{i\in L_{n}^{pos}}e^{-f_{i}\left(x_{n},\theta \right)}\right).$$

The LSEP loss function can be seen as a smooth approximation of hinge loss. However, it is unable to handle scenarios where the number of output labels is unknown, as it lacks an associated threshold. To address this limitation, Su et al. proposed ZLPR loss function in [27], and introduced a fixed threshold *T* on the model’s output scores for positive and negative labels. Specifically, the predicted label score for positive labels, $f_{i}\left(x_{n},\theta \right)$, where $i\in L_{n}^{pos}$, must be greater than *T*, while the predicted label score for negative labels, $f_{j}\left(x_{n},\theta \right)$, where $j\in L_{n}^{neg}$, must be less than *T*. The ZLPR loss can be written as

(3) $$\mathrm{ZLPR}=log\left(1+\sum_{j\in L_{n}^{neg}}e^{f_{j}\left(x_{n},\theta \right)}\sum_{i\in L_{n}^{pos}}e^{-f_{i}\left(x_{n},\theta \right)}+\sum_{j\in L_{n}^{neg}}e^{f_{j}\left(x_{n},\theta \right)-T}+\sum_{i\in L_{n}^{pos}}e^{T-f_{i}\left(x_{n},\theta \right)}\right).$$

Su et al. suggested T=0 and, thus, the above equations takes the following form:

(4) $$\mathrm{ZLPR}=log\left(1+\sum_{i\in L_{n}^{pos}}e^{-f_{i}\left(x_{n},\theta \right)}\right)+log\left(1+\sum_{j\in L_{n}^{neg}}e^{f_{j}\left(x_{n},\theta \right)}\right).$$

It is evident from the ZLRP loss function, as represented by Equation (4), that all labels are treated uniformly. As a result, it lacks an effective mechanism for handling rare labels, which are challenging to classify in the presence of majority labels. Inspired by the focal loss [11], we propose a label score scaling strategy for the ZLPR loss function. This strategy enhances the model’s ability to focus on hard examples by increasing their contribution to the loss function while reducing the contribution of well-classified examples. To achieve this, a temperature parameter τ is introduced in the ZLPR function with the purpose to scales the label scores. As a result, the loss function focuses on rare labels and addresses the class imbalance effectively. We refer to the modified ZLPR function as Focal ZLPR, abbreviated as FZLPR. Mathematically, we can write it as

(5) $$\mathrm{FZLPR}=log\left(1+\sum_{i\in L_{n}^{pos}}e^{\left(-f_{i}\left(x_{n},\theta \right)/\tau \right)}\right)+log\left(1+\sum_{j\in L_{n}^{neg}}e^{\left(f_{j}\left(x_{n},\theta \right)/\tau \right)}\right).$$

**Figure 1:**
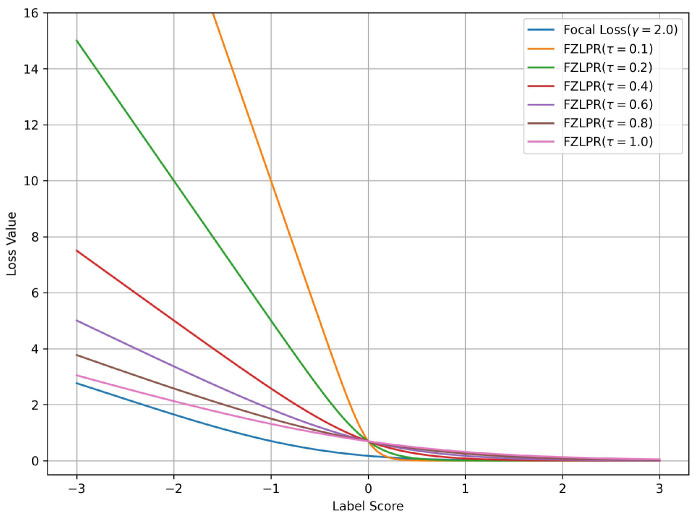
Loss values for the focal and ZLPR loss functions for a positive label score. The label score is greater than 0 for a well-classified example and is less than 0 for a misclassified example. Best viewed on a color display.

It is important to note that the Equation (5) is a generalization of the ZLPR function. To demonstrate the effectiveness of the label score scaling strategy in the FZLPR function, we analyze the impact of varying the temperature parameter τ on a single positive label and plot the corresponding loss values against the label scores in Figure 1. Since FZLPR treats both positive and negative labels similarly, this analysis applies to negative labels as well. Additionally, for reference, we include the focal loss with the recommended parameter setting (γ=2.0) in the same plot. Now, we consider the following cases corresponding to different regions in Figure 1:

- When the positive instance is correctly classified with high probability i.e., the label score $f_{i}\left(x_{n},\theta \right)\gg 0,i\in L_{n}^{pos}$, the weighting by τ causes its contribution to the loss function to become zero. This means the model does not focus on such instances, as their gradient is zero.
- When the positive instance is correctly classified but with low probability i.e., the label score $f_{i}\left(x_{n},\theta \right)\approx 0,i\in L_{n}^{pos}$, the weighting by τ ensures that its contribution is included in the loss function and its gradient flows during training.
- When the positive instance is incorrectly classified i.e., the label score $f_{i}\left(x_{n},\theta \right)<0$, $i\in L_{n}^{pos}$, the weighting by τ significantly increases its contribution to the loss function, ensuring that the model focuses on such instances and allowing a substantial gradient flow during training.

The results indicate that, in general, FZLPR behaves similarly to focal loss, reducing focus on well-classified examples while increasing the contribution of misclassified examples, which, in our case, are typically rare labels. Furthermore, misclassified examples incur a higher penalty compared to focal loss. The temperature parameter τ plays a critical role in scaling the label scores. In this work, τ is empirically determined as discussed in Section 4.

### 3.2. Transfer Learning

**Figure 2:**
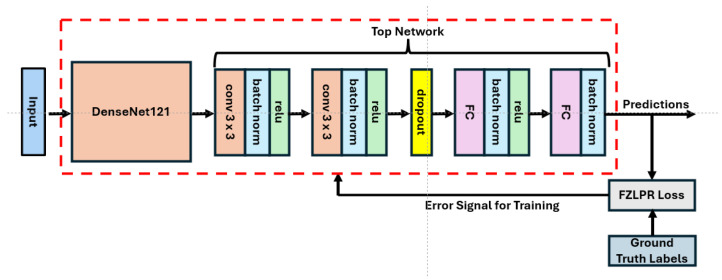
Architecture of our proposed model. The error signal generated by the loss function is used for training the model.

As stated in the introduction, transfer learning is a widely used technique for classifying thoracic diseases in CXR images. However, various sophisticated CNN architectures have been proposed in the literature for image classification. Among them, densely connected convolutional networks (DenseNets) are quite promising as they have shown remarkable performance on various vision tasks including image classification, object detection, semantic segmentation, etc. [22,44]. In this work, we have adopted DenseNet121 (pretrained on ImageNet-1K), which is a 121-layer DenseNet model, and have employed the standard transfer learning technique to tune it on the NIH Chest X-ray14 benchmark dataset. The architecture of our model, which includes DenseNet121 and a custom top network, is shown in Figure 2. The loss function computes the error signal that is used for training. The details of DenseNet121 and the custom network are presented below. Moreover, the implementation details are presented in Section 4.2.

The input block of DenseNet121 consists of a single convolutional layer with a kernel size of 7 × 7, a stride of 2, and 64 filters, followed by batch normalization and the ReLU activation function. This block processes a 224 × 224 × 3 image as input. Its output is then passed to a max-pooling layer with a size of 2 × 2 and a stride of 2. The remainder of the model consists of four dense blocks and three transition blocks, arranged in an alternating sequence. Each dense block contains a specific number of convolutional blocks, with each convolutional block incorporating two convolutional layers. The first layer employs a 1 × 1 kernel, while the second uses a 3 × 3 kernel. The output of the second convolutional layer within a dense block is concatenated with its inputs. This enables the architecture to reuse feature maps from previous layers to construct rich feature representations and facilitate efficient gradient flow. Each transition block is composed of a single convolutional layer with a 1 × 1 kernel, designed with the objective to reduce the number of feature maps from the preceding dense block. Batch normalization and the ReLU activation function are also used in both dense and transition blocks along with the convolutional layers. The final dense block yields 1024 feature maps, each one of size of 7 × 7 pixels, which are then passed to a custom top network rather than the default one.

It is important to note that images from the ImageNet-1K dataset and CXR datasets have fundamental differences as they belong to different domains. As a result, we believe that the default top network (a global pooling layer and a final classification layer) is not optimal for generating a rich representation of CXR images. To overcome this limitation, our custom top network is designed by stacking two convolutional layers, a dropout layer with dropout rate of 0.1 for regularization, and two dense layers. Both convolutional layers use 3 × 3 kernels with a stride of 1, followed by batch normalization layers and ReLU activations. The number of filters in the convolutional layers is set to 256 and 128, respectively. This configuration yields a 1152-dimensional feature vector for each input image, which is then fed to the two dense layers composed of 64 and 14 hidden units, respectively. The output of the first dense layer is passed through a batch normalization layer and a ReLU activation function before feeding it to the second dense layer that acts as the final classification layer. Additionally, batch normalization is applied to the output of the final classification layer to ensure stable training. The number of parameters in DenseNet121 and the custom top network is approximately 7.03 million and 2.73 million, respectively.

## 4. Experimental Results

This section presents the experimental results of our proposed methodology on the NIH Chest X-ray14 benchmark dataset. We also provide details on the training methodology and implementation, along with a comprehensive analysis of the results. Finally, a comparison with existing methods is presented.

### 4.1. Chest X-Ray Dataset and Evaluation Metric

**Figure 3:**
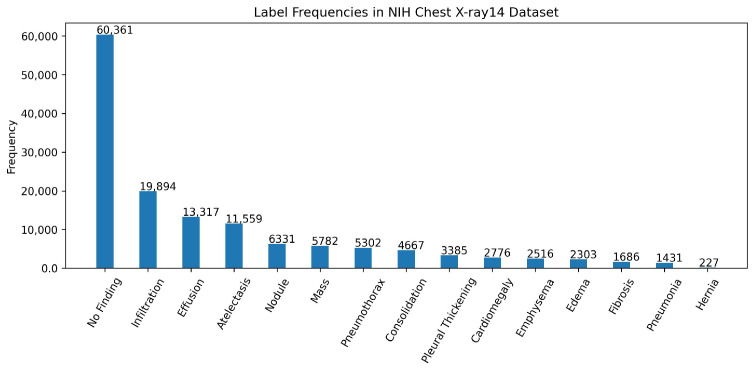
Label frequencies in the NIH chest x-ray14 benchmarks dataset. Note that the labels are sorted according to their frequencies.

**Figure 4:**
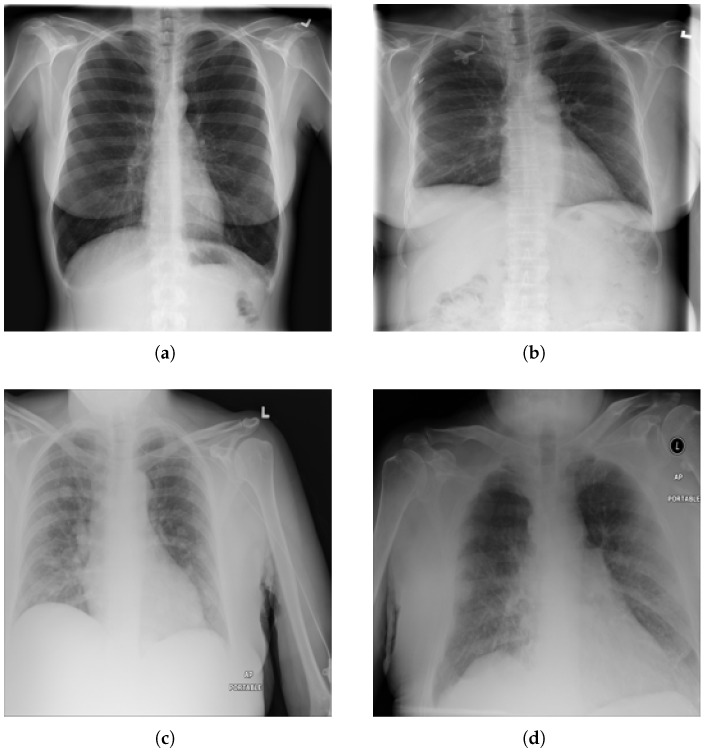
Examples from NIH chest X-ray14 datasets with labels: (**a**) No Finding, (**b**) Fibrosis. (**c**) Mass, and (**d**) Edema, Pleural Thickening, and Pneumonia.

We have used the NIH Chest X-ray14 benchmark dataset [4], which is a well-known and widely used benchmark dataset. It contains a total of 112,120 frontal CXR images from 30,805 patients in Digital Imaging and Communications in Medicine (DICOM) format. For research purposes, the dataset is also made available in PNG format where each image has a resolution of 1024 × 1024 pixels. Out of the total, 86,524 images are allocated for training, and the remaining 25,596 are used for evaluation. There is no overlap in patients between the training and test datasets. The dataset includes 51,759 CXR images with 14 different disease labels in a multi-label format, meaning a single CXR image can exhibit more than one disease. Additionally, there are 60,361 CXR images of normal patients. The label frequencies are shown in Figure 3, which also indicates the class imbalance problem associated with the dataset and making it a challenging task. Moreover, some examples from the dataset along with the findings are shown Figure 4. For training purposes, all images were downscaled from their original resolution to 256 × 256 pixels using bicubic interpolation. Moreover, we split the training set randomly to create a validation set of 8652 images using a 90:10 ratio. Labels are coded using a multi-hot encoding scheme. For evaluation, we employ the standard performance metric proposed by Wang et al. [4], which is widely adopted by researchers for the NIH Chest X-ray14 dataset. Specifically, the area under the receiver operating characteristic curve (AUC) is computed for each class in the MLC.

### 4.2. Training Methodology and Implementation Details

As mentioned in Section 3, we employ transfer learning using a pretrained DenseNet121 model. The training process consists of two stages. In the first stage, the DenseNet121 model, serving as the base model, remains frozen while the top network, composed of convolutional and dense layers, is trained. In the second stage, both the base model and the top network are trained together. In both stages, we use the AdamW optimizer (Adam with decoupled weight decay) [45]. Training is conducted using the mini-batch stochastic gradient descent algorithm with backpropagation, where the mini-batch size is set to 64. The learning rates are set to $10^{-3}$ and $10^{-5}$ for the first and second stages, respectively. The higher learning rate in the first stage facilitates adaptation of the top network using the generic features of the pretrained base model, while the lower learning rate in the second stage refines the complete model for the disease classification task at hand. Additionally, cosine annealing [46] is applied in the second stage to decay the learning rate to a minimum value of $10^{-7}$, starting from an initial value of $10^{-5}$. The weight decay is set to $4\times 10^{-3}$. The top network is trained for a total of five epochs in the first stage and 20 epochs in the second stage. These values were determined through early stopping in our initial experiments and subsequently fixed for all future experiments. Mini-batch preprocessing involves normalization using the mean and standard deviation of the ImageNet dataset. Furthermore, as described in [5], random augmentations are applied during batch formation, including random horizontal flipping, random rotations (±0.4 radians), and random cropping to 224 × 224 pixels from an original image size of 256 × 256 pixels.

Our computing platform consists of a desktop PC running Ubuntu 20.04, equipped with a single Nvidia GeForce RTX 1080Ti GPU with 11 GB of memory, an Intel^®^ Core™ i7-4470 processor, and 24 GB of RAM. We use Google TensorFlow [47] for the implementation of DenseNet121, the proposed top network, and the loss function. The total training time for our model is 186 min when using BCE and focal loss, whereas it increases to 189 min with ZLPR and FZLPR.

### 4.3. Results and Discussion

In this section, we present various experimental results of our trained DenseNet121 model on the NIH Chest X-ray14 dataset. Firstly, we analyze and compare the performance of the model using different loss functions. Four DenseNet121 models were trained using different loss functions, BCE, focal loss, ZLPR, and our proposed FZLPR with τ=0.2, following the training methodology detailed in the previous section. An ablation study on the impact of the hyperparameter τ is included in the next section. We compare the performance of these four models based on their average AUC scores on the validation set, as listed in Table 1. It is observed that the model trained with FZLPR (τ=0.2) outperforms all others, achieving an average AUC score of 83.44% on the validation set. The model trained with focal loss ranks second, while the model trained with BCE loss records the lowest average AUC score of 80.82%. To comprehensively compare the performance of these four models on the official test split of the NIH Chest X-ray14 dataset, we summarize the individual and average AUC scores obtained across 14 disease labels in Table 2. This follows established practices for reporting test set results in the field, as presented in previous research works [4,5,6]. The best and runner-up for each label is shown in bold and underlined text, respectively. It can be observed that both FZLPR and focal losses emphasize the correct prediction of rare labels including Hernia, Pneumonia, Fibrosis, Emphysema, Cardiomegaly, etc., while maintaining comparable AUC scores on the majority classes. FZLPR attained the best or runner-up positions for eight disease labels, while focal loss achieved the best or runner-up position for 12 disease labels. However, on average, the FZLPR loss outperforms focal, ZLPR, and BCE losses and achieves an average AUC of 80.45%.

**Figure 5:**
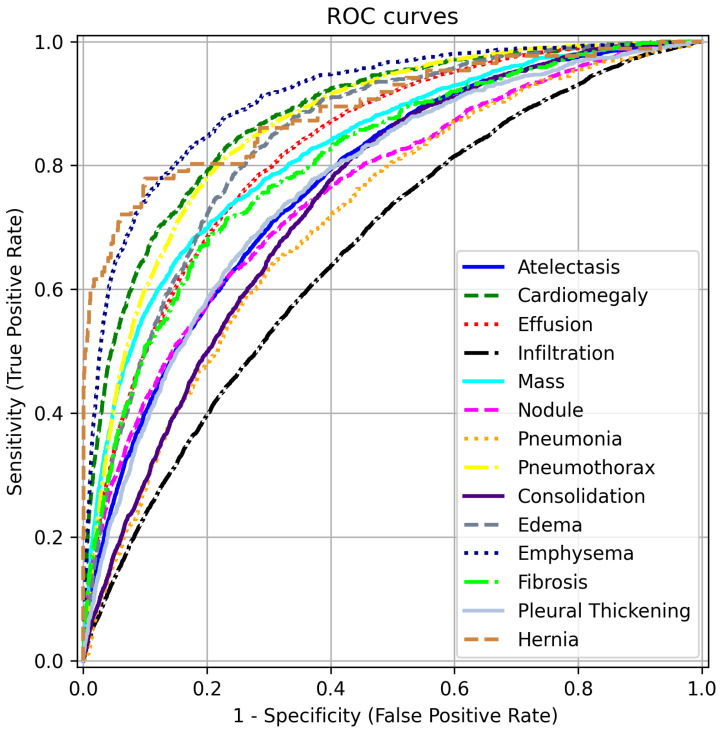
ROC curves for different disease labels using FZLPR (τ=0.2). Best viewed on a color display.

To complement the AUC scores in Table 2, we present ROC curves for each disease label in Figure 5. These curves are generated by varying the threshold applied to the label scores of our model, trained with FZLPR (τ=0.2), and computing sensitivity and specificity metrics. It can be observed that the model has good discrimination ability for all disease labels. In particular, the model achieves an AUC score of 80% or more for labels Including Emphysema, Hernia, Cardiomegaly, Pneumothorax, Edema, Effusion, Mass, And Fibrosis. This indicates that the model has significant clinical utility in distinguishing these pathologies from their absence. However, lower AUC scores are obtained for Infiltration and Pleural Thickening, which indicates that the model predictions must be accompanied by additional diagnostic information to be used in clinical settings.

Secondly, inspired by [6], we apply test-time augmentations (TTA) to the CXR images in the test set, as they are known to improve the generalization of the model. Specifically, we use horizontal flipping and a ±10% zoom transformation. Both the original and augmented images are fed into the model (DenseNet121+ FZLPR (τ=0.2)). The model’s outputs are then averaged to generate enhanced predictions. The impact of these transformations is summarized in Table 3. Notably, both augmentations contribute to improved model predictions. The average AUC increases by 0.25% when using only horizontal flipping, while employing both augmentations leads to a 0.51% increase.

### 4.4. Ablation Study

**Figure 6:**
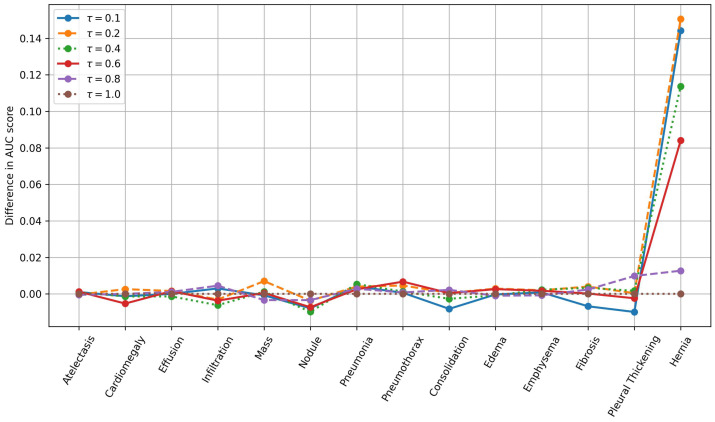
Variation in AUC scores across diseases labels for different values of τ. The baseline is chosen as τ=1.0 and differences in AUC scores are plotted. Best viewed on a color display.

We conduct an ablation study to analyze the impact of the hyperparameter τ in the FZLPR loss function. The value of τ is varied between 0.1 and 1.0, using the discrete set {0.1,0.2,0.4,0.6,0.8,1.0}. The individual AUC scores for different disease labels in the Chest X-ray14 dataset, along with the average AUC score, are presented in Table 4. The best and runner-up for each label is shown in bold and underlined text, respectively. Moreover, we present the variation in AUC scores across different disease labels for various values of τ, while fixing the baseline at τ=1.0, as shown in Figure 6. It is to be noted that the difference in AUC scores is plotted in the figure. The results indicate that τ=0.2 achieves the highest AUC scores across six labels, outperforming all other choices. This is followed by τ=0.8, which attains the best AUC scores on three labels. If both the best and runner-up positions are considered, τ=0.2 performs well across nine labels, followed by τ=0.4 and τ=0.8, with each performing across five labels. Overall, as τ decreases from 1.0 to 0.2, the average AUC score improves from 79.21% to 80.45%, with the highest average AUC achieved at τ=0.2. However, at τ=0.1, the average AUC score decreases by 0.39% compared to τ=0.2. In terms of AUC scores across diseases labels, significant gains are achieved using τ=0.2 compared to baseline (τ=1.0), particularly for the ‘Hernia’ label, as shown in Figure 6. In summary, these results indicate that when τ is small or large enough, the performance decreases, as it leads to extreme scaling of the label scores, which hinders the model’s effectiveness.

### 4.5. Comparison with Existing Methods

We now compare the experimental results of our proposed approach with existing methods. Six techniques from the literature are considered for comparison, including the foundational work of Wang et al. [4], which introduced the NIH Chest X-ray14 dataset. These methods were selected based on the diversity of their methodologies. The AUC scores across different disease labels are summarized in Table 5. To facilitate comparison, we have also included the difference ($\Delta_{AUC}$) between the AUC of our approach and the best-performing method. Moreover, the best AUC is highlighted in bold. It is important to note that Kufel et al. [28], like many other researchers in the field, do not use the official split of the dataset and achieve the highest average AUC score among all methods by fine-tuning an EfficientNet model. Therefore, such comparisons must be interpreted with caution. All other methods, including ours, in Table 5 use the official split. It is to be noted that our method employs a ranking loss, in contrast to all other methods, which use either BCE or its variants for model training or fine-tuning. Our approach outperforms the foundational work of Wang et al. [4], which employs transfer learning to fine-tune a ResNet50 model. Their fine-tuned ResNet50 achieved an average AUC of 74.51%. Moreover, our method slightly outperforms those of Guendel et al. [33] and Baltruschat et al. [30]. It is noteworthy that Gundel et al. use high-resolution images (1024 × 1024 pixels) to fine-tune a DenseNet121 model and Baltruschat et al. integrate non-image data alongside high resolution CXR images (448 × 448 pixels) to train a custom ResNet model. The inclusion of additional information in these two approaches helped them achieve better AUC scores. However, in contrast, our method using 224 × 224 pixels image as input with no additional (non-image) data outperforms these two approaches. Gaun et al. [20] employ an attention module to assign weights to different spatial positions of the DenseNet121 feature maps, in addition to fine-tuning, and achieve a slightly higher average AUC score—just 0.63% above that of our approach. The ensemble model proposed by Kotana et al. [5], composed of InceptionV3, ResNet50, and DenseNet121, outperforms all other methods including ours, with an average AUC of 83.07%. It must be noted that their ensemble was pretrained on additional data from MIMIC-CXR dataset before fine-tuning on the NIH Chest X-ray14 dataset. Moreover, their ensemble model comprises 57.3 million parameters, which is significantly higher compared to our DenseNet121 model, which contains just 9.76 million parameters. When analyzing the AUC scores of individual disease labels, our method exhibits a maximum $\Delta_{AUC}$ of 6.78% for the Consolidation label, while the minimum $\Delta_{AUC}$ of 2.53% is observed for the Cardiomegaly label. However, in terms of average AUC, our approach falls short of the best-performing method [28] by just 3.31%. Based on this comparison with existing methods, we conclude that our proposed approach achieves competitive experimental results.

### 4.6. Qualitative Results

**Figure 7:**
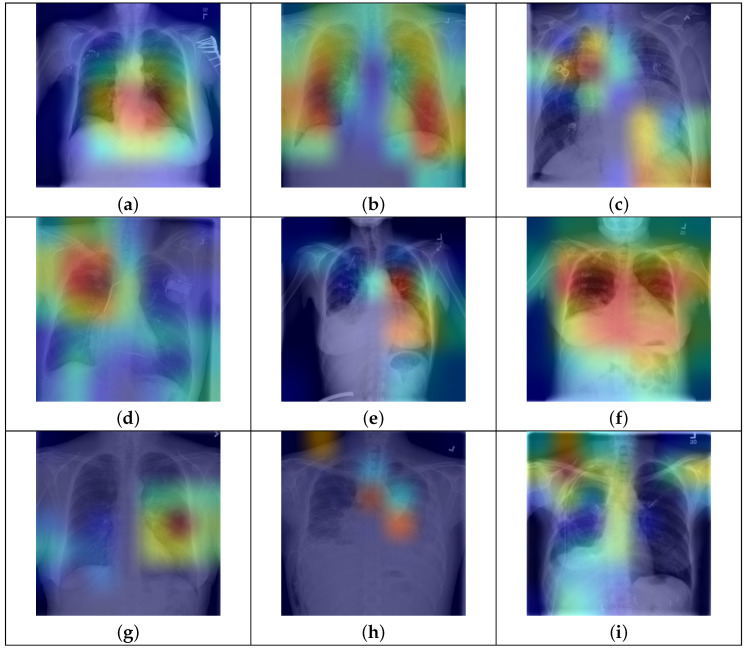
Qualitative results on the test set of NIH Chest X-ray14 dataset. Each CXR image is blended with its respective Grad-CAM and shows the high activity regions in red. Ground truth and predicted labels are mentioned for each test CXR image. (**a**) Ground Truth: Hernia Predicted: Hernia; (**b**) Ground Truth: Nodule Predicted: Nodule; (**c**) Ground Truth: Mass Predicted: Mass; (**d**) Ground Truth: No Finding Predicted: No Finding; (**e**) Ground Truth: Cardiomegaly, Effusion Predicted: Cardiomegaly, Effusion; (**f**) Ground Truth: Atelectasis, Effusion, Infiltration Predicted: Atelectasis, Effusion, Infiltration. (**g**) Ground Truth: Nodule, Fibrosis Predicted: Nodule; (**h**) Ground Truth: Effusion, Mass, Emphysema Predicted: Effusion, Mass; (**i**) Ground Truth: Pleural Thickening Predicted: Pneumothorax.

Finally, we present qualitative results of thoracic disease classification. To determine predictions for test CXR images, we set the label score threshold to 0. Ground truth and predicted labels for nine test CXR images are shown in Figure 7. Additionally, we employ the Grad-CAM technique to highlight the active regions of feature maps that significantly contribute to predictions. The feature maps from the “conv5” block of our model are used to generate Grad-CAMs. CXR images are then blended with the respective Grad-CAMs to visualize these regions, with high-activity areas displayed in red. Our model’s predictions align with the ground truth labels for both single and multiple disease cases, as seen in Figure 7a–f. It can be observed that the model highlights regions likely associated with disease, as indicated by high-activity areas in the Grad-CAM images. In the case labeled as “No Finding”, the model primarily focuses on regions of the lung that appear healthy. The third row highlights instances where the model fails to predict accurately. Specifically, in Figure 7g,h, the model could not predict one more disease label. In Figure 7i, we can observe that the model fails to accurately identify the disease-affected areas and, therefore, the prediction is incorrect.

## 5. Conclusions and Future Works

In this article, we address the challenging task of automatic thoracic disease classification in CXR images, while also handling the inherent class imbalance. Our methodology is based on transfer learning, utilizing a pretrained DenseNet121 model, which we fine-tune on a CXR image dataset. To effectively handle class imbalance, we propose FZLPR loss—a modification of ZLPR loss—that scales model output scores to focus on hard examples during training. The results are presented on the NIH Chest X-ray14 benchmark dataset, a widely recognized and challenging dataset. A comparison of FZLPR loss with other loss functions, such as BCE and focal losses, demonstrates its effectiveness in addressing class imbalance. Furthermore, we find that test-time augmentations enhance model performance. A comparison with existing methods reveals that our model, trained using FZLPR loss, achieves a performance comparable to that of state-of-the-art approaches.

For future work, we plan to expand our experimentation on a dataset with a broader range of disease labels, as the current evaluation is conducted on the NIH Chest X-ray14 dataset containing 14 disease labels only. To achieve this, the MIMIC-CXR-JPG v2.1.0 dataset can be used, which includes 40 disease labels and also exhibits a long-tail distribution of labels. Additionally, we aim to assess the performance of FZLPR on multi-label classification tasks in fields other than medical image analysis. To achieve this, we plan to utilize datasets such as MS-COCO [48], a benchmark dataset for object detection, and AAPD [49], a benchmark dataset for text classification.

## Figures and Tables

**Table 1:** Evaluation of models trained with different loss functions based on average AUC scores across different disease labels in the validation set.

Loss Function	Validation Set
BCE	0.8082
Focal Loss	0.8312
ZLPR	0.8238
FZLPR (τ=0.2)	0.8344

**Table 2:** Evaluation of models trained with different loss functions based on AUC scores across different disease labels in the test set. The best AUC scores are shown in bold text while the runner-up is shown in underlined text.

Disease Label	BCE	Focal Loss	ZLPR	FZLPR (τ=0.2)
Atelectasis	0.7716	**0.7770**	0.7733	0.7729
Cardiomegaly	0.8742	**0.8834**	0.8761	0.8787
Effusion	0.8270	**0.8273**	0.8250	0.8266
Infiltration	**0.6831**	0.6765	0.6686	0.6655
Mass	0.8241	0.8246	0.8177	**0.8247**
Nodule	0.7697	**0.7723**	0.7619	0.7582
Pneumonia	0.7042	**0.7290**	0.7118	0.7158
Pneumothorax	0.8628	0.8599	0.8593	**0.8639**
Consolidation	**0.7458**	0.7437	0.7442	0.7447
Edema	0.8414	**0.8456**	0.8374	0.8403
Emphysema	0.9072	**0.9112**	0.9057	0.9077
Fibrosis	0.8004	**0.8083**	0.8018	0.8058
Pleural Thickening	0.7707	**0.7723**	0.7666	0.7670
Hernia	0.7276	0.7740	0.7406	**0.8911**
Average	0.7936	0.8004	0.7921	**0.8045**

**Table 3:** AUC scores across different disease labels in the test set using the test time augmentations. HF: horizontal flip. Z: zoom (±10%).

Disease Label	Test Image	Test Image + HF	Test Image + HF + Z
Atelectasis	0.7729	0.7754	0.7785
Cardiomegaly	0.8787	0.8818	0.8857
Effusion	0.8266	0.8287	0.8289
Infiltration	0.6655	0.6688	0.6848
Mass	0.8247	0.8301	0.8313
Nodule	0.7582	0.7622	0.7687
Pneumonia	0.7158	0.7160	0.7174
Pneumothorax	0.8639	0.8677	0.8670
Consolidation	0.7447	0.7462	0.7472
Edema	0.8403	0.8428	0.8436
Emphysema	0.9077	0.9096	0.9066
Fibrosis	0.8058	0.8101	0.8125
Pleural Thickening	0.7670	0.7671	0.7670
Hernia	0.8911	0.8909	0.8952
Average	0.8045	0.8070	0.8096

**Table 4:** Ablation study on the impact of τ in the FZLPR loss function. The AUC scores across different disease labels in the Chest X-ray14 dataset are listed. The best AUC scores are shown in bold text while the runner-up is shown in underlined text.

Disease Label	τ=0.1	τ=0.2	τ=0.4	τ=0.6	τ=0.8	τ=1.0
Atelectasis	0.7743	0.7729	0.7741	**0.7745**	0.7728	0.7733
Cardiomegaly	0.8748	**0.8787**	0.8747	0.8708	0.8761	0.8761
Effusion	0.8250	**0.8266**	0.8236	0.8265	0.8261	0.8250
Infiltration	0.6716	0.6655	0.6624	0.6649	**0.6731**	0.6686
Mass	0.8169	**0.8247**	0.8189	0.8182	0.8143	0.8177
Nodule	0.7540	0.7582	0.7521	0.7546	0.7585	**0.7619**
Pneumonia	0.7159	0.7158	**0.7172**	0.7143	0.7146	0.7118
Pneumothorax	0.8599	0.8639	0.8606	**0.8660**	0.8601	0.8593
Consolidation	0.7360	0.7447	0.7415	0.7446	**0.7464**	0.7442
Edema	0.8371	**0.8403**	0.8366	0.8400	0.8364	0.8374
Emphysema	0.9065	0.9077	**0.9080**	0.9074	0.9049	0.9057
Fibrosis	0.7950	**0.8058**	0.8051	0.8020	0.8042	0.8018
Pleural Thickening	0.7567	0.7670	0.7683	0.7642	**0.7764**	0.7666
Hernia	0.8848	**0.8911**	0.8543	0.8246	0.7533	0.7406
Average	0.8006	**0.8045**	0.7998	0.7980	0.7941	0.7921

**Table 5:** Comparison with existing methods using AUC score across different disease labels. The best AUC score is shown in bold text. $\Delta_{AUC}$ is the difference between the AUC of our approach and the best-performing method.

Disease Label	Wang et al. [4]	Gundel et al. [33]	Guan et al. [20]	Baltruschat et al. [30] ^1^	Kufel et al. [28] ^2^	Kotana et al. [5] ^3^	Proposed with TTA	Approach $\Delta_{\mathrm{AUC}}$
Atelectasis	0.7003	0.7670	0.7810	0.7630	0.8170	**0.8323**	0.7785	−0.0538
Cardiomegaly	0.8100	0.8830	0.8830	0.8750	**0.9110**	0.9014	0.8857	−0.0253
Effusion	0.7585	0.8280	0.8310	0.8220	0.8790	**0.8915**	0.8289	−0.0626
Infiltration	0.6614	0.7090	0.6970	0.6940	**0.7160**	0.6489	0.6848	−0.0312
Mass	0.6933	0.8210	0.8300	0.8200	0.8530	**0.8676**	0.8313	−0.0363
Nodule	0.6687	0.7580	0.7640	0.7470	0.7710	**0.7773**	0.7687	−0.0086
Pneumonia	0.6580	0.7310	0.7250	0.7140	**0.7690**	0.7465	0.7174	−0.0516
Pneumothorax	0.7993	0.8460	0.8660	0.8400	**0.8980**	0.8651	0.8670	−0.0310
Consolidation	0.7032	0.7450	0.7580	0.7490	**0.8150**	0.8123	0.7472	−0.0678
Edema	0.8052	0.8350	0.8530	0.8460	**0.9080**	0.9040	0.8436	−0.0644
Emphysema	0.8330	0.8950	0.9110	0.8950	**0.9350**	0.8918	0.9066	−0.0284
Fibrosis	0.7859	0.8180	**0.8260**	0.8160	0.8240	0.8076	0.8125	−0.0135
Pleural Thickening	0.6835	0.7610	0.7800	0.7630	0.8120	**0.8208**	0.7670	−0.0538
Hernia	0.8717	0.8960	0.9180	**0.9370**	0.8900	0.8628	0.8952	−0.0418
Average	0.7451	0.8066	0.8159	0.8058	**0.8427**	0.8307	0.8096	−0.0331

^1^ Use additional non-image data. ^2^ Use a non-official split. ^3^ An ensemble method and use additional training data from MIMIC-CXR dataset.

## Data Availability

NIH Chest X-ray14 dataset is a publicly available benchmark dataset. It can be found here: https://nihcc.app.box.com/v/ChestXray-NIHCC (accessed on 15 June 2024).
